# The Effect of Ambiguity Awareness on Second Language Learners’ Prosodic Disambiguation

**DOI:** 10.3389/fpsyg.2020.573520

**Published:** 2020-09-18

**Authors:** Yuanyuan Zhang, Hongwei Ding

**Affiliations:** ^1^ Speech-Language-Hearing Center, School of Foreign Languages, Shanghai Jiao Tong University, Shanghai, China; ^2^ MARCS Institute, University of Western Sydney, Penrith, NSW, Australia

**Keywords:** ambiguity awareness, prosody, L2, syntactic ambiguity, prepositional-phrase attachment

## Abstract

Three tasks were reported to examine the effect of ambiguity awareness on Chinese-speaking English learners’ use of prosody in resolving prepositional-phrase attachment ambiguity. In the first (Task 1) and second (Task 2) tasks, listeners were not informed of the syntactic ambiguity. In the third task (Task 3), listeners were given the specific information about syntactic ambiguity. The analysis of the overall accuracy rate showed that before receiving specific information about syntactic ambiguity, learners did not detect the ambiguity within the structure and tended to interpret the sentence in a “good-enough” heuristic to reduce the computational burden. After being aware of the syntactic ambiguity, they could use prosodic cues to resolve the ambiguity. However, the finding that the learners reversed their parsing bias from verb phrase attachment (VP-attachment) toward noun phrase attachment (NP-attachment) indicated their difficulty in integrating prosodic information to syntactic structure efficiently. The analysis of individual accuracy rate demonstrated learners’ individual variations in using prosodic cues. The result suggests that learners’ failure to use prosodic cues may be attributed to a lack of ambiguity awareness and difficulty in information integration, rather than their low sensitivity to prosodic cues.

## Introduction

Syntactic ambiguity has been widely studied in both native (L1) and second languages (L2) to investigate sentence parsing strategies. The parser needs to coordinate multiple domains of information to resolve the ambiguity. Therefore, syntactic ambiguity resolution can reveal not only the parser’s attachment preference for a given syntactic structure and its recovery from initial misinterpretations (Frazier and Rayner, 1982; Clifton et al., 1991; Anderson and Carlson, 2010), but also the multiple constraints that affect sentence parsing strategies (Beach, 1991; MacDonald et al., 1994; Trueswell et al., 1994; Tanenhaus et al., 1995; Stirling, 1996; Baum and Dwivedi, 2003; Snedeker and Trueswell, 2003). It is found that the parser’s ability to resolve syntactic ambiguity involves not only sentence parsing strategy but also ambiguity awareness that has been categorized in metalinguistic awareness (Tunmer and Bowey, 1984; Zipke et al., 2009). The serial parsing strategy, or the garden-path model, proposes that processing difficulty emerges when the current analysis is contradicted with the subsequent input (Frazier, 1978, 1987; Frazier and Rayner, 1982; Ferreira and Henderson, 1991). By contrast, the parallel parsing model, or the constraint-based model, holds that the parser’s processing difficulty results from the competition between multiple analyses (Spivey-Knowlton et al., 1993; MacDonald et al., 1994; Trueswell et al., 1994; Spivey-Knowlton and Sedivy, 1995; Tanenhaus et al., 1995; McRae et al., 1998). However, processing difficulty may also come from the possibility that the parser does not detect the ambiguity at all. Without ambiguity awareness, if the parser chooses the correct analysis, it will avoid a reanalysis of the structure; if an incorrect analysis is adopted, the disambiguating information may not trigger reanalysis (Slattery et al., 2013). The “good-enough” approach suggests that if the parser does not detect the ambiguity within the structure, it will analyze it in a “good-enough” heuristic and may quickly and efficiently create a superficial structure, which seems “good-enough” instead of a full analysis of the sentence (Ferreira et al., 2002). The parser holds the “good-enough” interpretation till the end of the sentence even after the disambiguating information becomes available (Ferreira et al., 2001, 2002; Ferreira and Patson, 2007; Slattery et al., 2013; Christianson, 2016). Therefore, to resolve syntactic ambiguity, the parser needs to detect the ambiguity within the structure before adopting an appropriate parsing strategy to fully analyze the structure.

**Figure 1:**
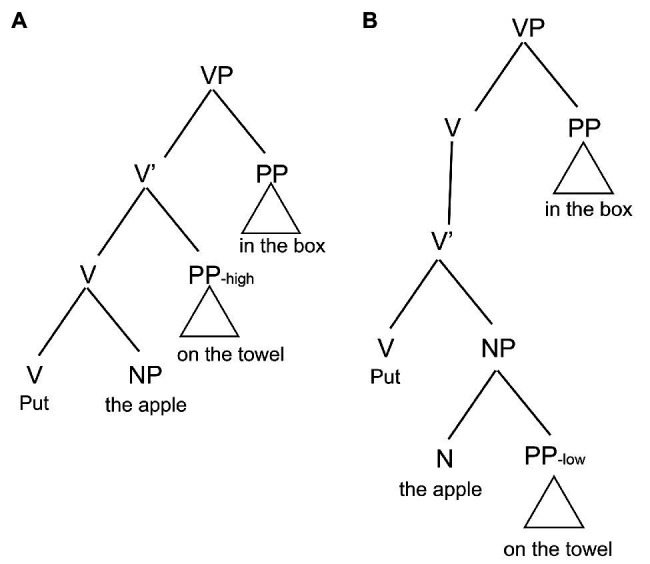
**(A)** Attach to the head of PP_-high_. **(B)** Attach to the head of PP_-low_.

Being different from written text, the message in spoken language is also conveyed through prosody, which refers to the suprasegmental properties of speech signal, including stress, rhythm, and intonation realized through varying the fundamental frequency (F0), duration, and amplitude (Cutler and Swinney, 1987). Prosody provides listeners with valuable information about word recognition, syntactic parsing, information structure of the utterance, and the speakers’ affective mode. However, the information conveyed in spoken language is transient because the speech signal loses rapidly, and the parser cannot refer back to the text as the sentence unfolds. Spoken language comprehension is consequently further burdened by the transient nature of speech signals. Prior research has shown that prosodic phrasing can affect listeners’ parsing decisions (see Cutler et al., 1997, for a review). Listeners can make use of the alignment of the prosodic boundary with the major syntactic boundary to determine the alternative interpretation of syntactic ambiguity and reduce their parsing bias (Lehiste, 1973; Price et al., 1991; Schafer, 1997; Tree and Meijer, 2000; Warren et al., 2000; Clifton et al., 2002; Snedeker and Trueswell, 2003; Kraljic and Brennan, 2005; Diehl et al., 2008; Nakamura et al., 2012). For instance, in early closure (EC)/late closure (LC) ambiguity, a prosodic boundary immediately after the initial phrase “whenever a bear is approaching the people…” can guide listeners toward the LC reading, avoiding the early garden-path effect “whenever a bear is approaching….” Moreover, if the prosodic boundary is absent or if it is inconsistent with the syntactic boundary, processing difficulty will increase (Pauker et al., 2011). In sentences with global ambiguity, such as the prepositional-phrase attachment (PP-attachment) ambiguity, the placing of the prosodic boundary can distinguish the two alternative interpretations of the ambiguity while keeping the whole structure intact. For example, a prosodic boundary immediately after “apple” or “towel” in “put the apple on the towel in the box” can direct listeners toward either the high verb phrase attachment (VP-attachment) or the low noun phrase attachment (NP-attachment) (Schafer, 1997; Kraljic and Brennan, 2005; Schafer et al., 2005; Snedeker and Yuan, 2008). The syntactic tree structures for the high VP-attachment and the low NP-attachment are respectively shown in Figures 1A,B. In the high VP-attachment, the first prepositional-phrase “on the towel” (PP1) is attached to “put” (VP); in the low NP-attachment, PP1 is attached to “the apple” (NP). Experiments with event-related potentials (ERPs) provide evidence that prosodic information can guide listeners’ interpretation at the initial stage of sentence processing (Steinhauer et al., 1999; Pannekamp et al., 2005; Pauker et al., 2011) and override their parsing preference induced by the lexical information, discourse context, and visual context (Altmann and Steedman, 1988; Britt, 1994; Spivey-Knowlton and Sedivy, 1995; Tanenhaus et al., 1995; van Berkum et al., 1999; Spivey et al., 2002). Therefore, the effect of prosody on sentence processing has been proven to be robust in L1 research.

Recent years have witnessed an increasing investigation into the L2 learners’ use of prosody in L2 processing. Earlier studies reported that older L2 learners were as likely as younger ones to attend to prosodic rather than syntactic information (Harley et al., 1995). Later studies showed that the effect of prosody is partial and related to the learners’ L1 background. For example, Chinese L2 learners were suggested to be less sensitive to prosodic cues than learners from other language groups (e.g., Mexican, German, French, Swedish, etc.; Ying, 1996). The effect of prosody is also found to be related to the learners’ learning experience. The second-semester college-level learners were less sensitive to prosodic cues than the fourth-semester learners (Dekydtspotter et al., 2008). Moreover, studies using ERPs found that Chinese learners of English showed different processing patterns from German learners and native English speakers, which might be due to their low proficiency (Nickels and Steinhauer, 2018). A further study found that L2 learners displayed different processing strategies even when their L1 and L2 had identical ambiguity and disambiguation patterns, suggesting that L2 learners’ parsing strategy has to be learned anew (Ip and Cutler, 2018). In addition, the learners might be less able to integrate prosodic information to other domains of information compared to native speakers (Nakamura et al., 2016, 2020). The above findings indicate that L2 learners’ ambiguity resolution can be constrained by linguistic and nonlinguistic information, and the L1–L2 differences in prosodic disambiguation can be attributed to a variety of factors, such as L2 proficiency, L1 background, low sensitivity to prosodic cues, and difficulty in information integration.

Most of the prior studies in L2 sentence processing have focused on reading tasks, finding that L2 learners have difficulty in recovering from misanalysis of the garden-path sentences, which might be due to their parsing deficits but not competence (Juffs and Harrington, 1996). That is to say, L2 learners have the ability to acquire the target grammatical knowledge, but not to use this knowledge properly to build L2 representations. They have more difficulties with the more complex structures (Roberts and Felser, 2011), and in integrating multiple information sources (Roberts et al., 2008), as has been proposed in the Interface Hypothesis that L2 learners experience more difficulties when sentence processing involves an integration of multiple information sources (Sorace and Serratrice, 2009). Some researchers argued in Shallow Structure Hypothesis (SSH) that L2 learners rely more on lexical, semantic, and pragmatic rather than syntactic information, and therefore, their parsing strategy is “shallower” than the native speakers, and is fundamentally different from that of the native speakers (Papadopoulou and Clahsen, 2003; Papadopoulou, 2005; Clahsen and Felser, 2006a,b). By contrast, studies with ERPs and other online measures have provided evidence that L2 learners may show the native-like processing patterns if their L2 proficiency is sufficiently high. The L1–L2 differences may be attributed to their L2 proficiency and cognitive resources (Just and Carpenter, 1992; Hopp, 2006, 2014; Steinhauer, 2006; Jackson and Dussias, 2009; Steinhauer et al., 2009; Roberts, 2012; Witzel et al., 2012; Reichle et al., 2016; Nickels and Steinhauer, 2018).

In L2 spoken language, sentence comprehension requires L2 learners to integrate prosodic information to syntactic information so that the sentence can be fully analyzed. However, one cannot expect L2 learners to fully understand the ambiguous sentences if they do not detect the ambiguity at all. Previous studies have reported that the learner’s failure to use prosodic cues may result from various factors, such as their low sensitivity to prosodic cues, difficulty in integrating multiple sources of information, L2 proficiency, etc. There is another possibility that the learners’ unawareness of the ambiguity within the structure leads to their failure to fully analyze the structure. The current study aims to investigate Chinese L2 English learners’ use of prosody in PP-attachment ambiguity resolution, with a focus on the effects of ambiguity awareness. Our assumption was that if the learners do not have ambiguity awareness to detect the syntactic ambiguity, they will not be able to fully analyze the ambiguous structure even though the disambiguating information is available. As a result, they tend to interpret the ambiguous structure in a “good-enough” heuristic, ignoring a deeper reanalysis of the structure that is consistent with the available prosodic cues, leading to their failure to use prosodic cues. However, if they still could not resolve syntactic ambiguity with prosody even after they were aware of the ambiguity, their analysis of the ambiguous sentences may be constrained by other factors, such as low sensitivity to prosodic cues and difficulty in information integration.

## Materials and Methods

The experiment included three tasks in which the accuracy rate was analyzed to determine the degree of participants’ understanding of the ambiguous sentences. Participants in the current task were not informed of the following task so that they would not try to remember what they heard and saw. Our target sentences were randomly selected from the spontaneously produced PP-attachment sentences by 10 native speakers so that L2 listeners’ processing of spontaneous speech can be investigated.

### Participants

Thirty adult Chinese-speaking learners of English (aged 18–21 years) from Shanghai took part in this experiment. They were native speakers of Chinese and had normal or corrected-to-normal vision and normal hearing. All were second-year undergraduate students and had passed college English test band 4 (CET-4). They had been learning English for about 10 years, but none of them had learned English in any English-speaking countries or majored in English. All participants received a small payment for their participation.

### Materials and Design

The materials included target and filler items. The target items with PP-attachment ambiguity were randomly selected from the 10 native American English speakers who participated in the previous speech production study (Zhang et al., 2018). For each speaker, only one pair of sentences was selected. All the sentences had the same structure (VP-NP-PP1-PP2), which was adopted from prior studies by Kraljic and Brennan (2005) and Tanenhaus et al. (1995). Within this structure, PP1 can be attached high to VP (VP-attachment) or low to NP (NP-attachment) by locating a pause immediately after NP or PP1, alternatively, as has been shown in Figures 1A,B. For instance, “Put the dog (PAUSE) in the basket on the mat” means “Put the dog in the basket that is on the mat” for VP-attachment, while “Put the dog in the basket (PAUSE) on the mat” means “Put the dog that is in the basket on the mat” for NP-attachment. However, in Chinese, PP modifiers always immediately precede the constituent they modify (Pan and Felser, 2011). The structural differences in PP-attachment between Chinese and English make PP-attachment ambiguity the best syntactic structure and Chinese learners of English the best subjects to test ambiguity awareness. The target sentences were produced by 10 different native American English speakers in a role-play game task. As has been revealed, all the utterances have been appropriately disambiguated by means of prosody supporting either VP-attachment or NP-attachment. Speakers inserted a pause immediately after NP or PP1 in VP-attachment or NP-attachment, respectively, lengthened the duration of the pre-boundary syllable, and reset pitch values at the boundary. Moreover, all the utterances have been correctly distinguished by the phonetically trained native confederate during the game playing (Zhang et al., 2018). We also constructed 15 filler items based on the target items to distract participants’ attention to the ambiguous structure. The filler items, which had a similar structure to the target ones, were unambiguous. One female native speaker who took part in the role-play game task produced the filler items. In total, 35 utterances were employed in this experiment.

**Figure 2:**
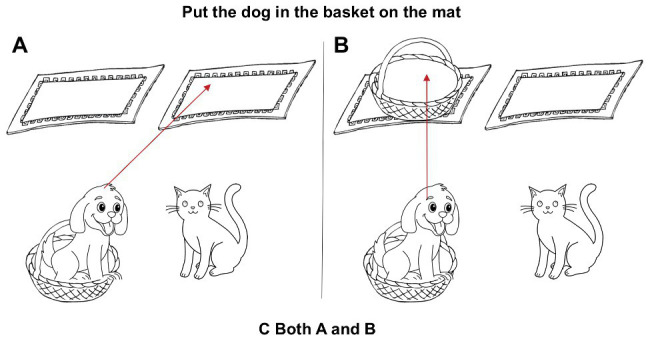
Picture display in Task 2.

The stimuli were grouped into two blocks with a mixed assignment of half VP-attachment and half NP-attachment of each pair in each block. Target and filler items were arranged in a pseudo-random order with at least one filler between two target items. The object to be moved is counterbalanced at the lower right or left side of the display, and the destination object is counterbalanced at the upper right or left side of the display. In Tasks 1 and 3, the sentences were presented through headphones and pictures on the computer monitor indicating the alternative interpretation of the ambiguous utterances. Participants needed to select one of the pictures that could best interpret the meaning of the sentences that they heard. In Task 2, the sentences were presented with pictures on the monitor instead. Participants were asked to select one or two pictures that corresponded to the possible meaning of the sentences. The picture display in Task 2 was given in Figure 2.

### Procedure

All tasks were carried out on a laptop *via* E-prime 2.0 program (Psychology Software Tool Inc., United States). Task 1 was completed in the 1st week, and Tasks 2 and 3 were conducted 1 week later to mitigate repetition effects (Pell, 2005). Participants were tested individually in a quiet room on the university campus. Before the experiment, participants read the experiment procedure on the monitor, and then the experimenter explained to them orally. Participants were told that they would listen to a few sentences in Task 1. For each sentence, there were two pictures on the screen. Their task was to listen to the sentences and look at the pictures carefully, and then to select one of the pictures that corresponded to the meaning of the sentence that they heard. They should give their response as soon as they understood the sentence. Before each trial, participants would see a red cross (i.e., “+”) in the middle of the screen, and meanwhile hear a beep *via* the headphones (AKG K240MkII), which would last for 500 ms. Then each sentence would be played once, and the picture display would remain on the screen until they gave their response by pressing the corresponding button. One week later, participants were told to do Task 2 in which the sentences were the same as those in Task 1 except that they were presented on the monitor with pictures. They needed to read the sentence above the picture display, and select one or two pictures that could interpret the possible meaning of the sentence. The pictures and sentences would remain on the screen until they gave their response. After Task 2, the experimenter interviewed the participants about the ambiguity. All participants reported that they did not know there might be two alternative interpretations for this structure before this experiment, but one of them realized the syntactic ambiguity in Task 1, and four additional participants realized the syntactic ambiguity in Task 2. The experimenter then gave them the specific information about the syntactic ambiguity but did not provide any prosodic strategies to disambiguate it. Then after a short rest, Task 3 was conducted. Participants were told that Task 3 was the same as Task 1. The overall experiment was preceded by a familiarization phase, in which participants were presented with the isolated objects and their English names that would appear in the experiment, and completed six practice trials. During the whole experiment, the participants did not receive any feedback on how to disambiguate these sentences with prosodic means; therefore the training effects were minimized.

### Data Analysis

The data analyses focused on the participants’ accuracy score in each task to investigate L2 learners’ overall understanding of the sentences, and the individual’s accuracy score across three tasks to explore the individual’s differences in their understanding of the sentences before and after they were informed of the ambiguity. Participants’ responses to the practice/filler items were excluded from analyses. In addition, two participants’ responses were also discarded since they took part in Task 1 but not in Tasks 2 and 3. In total, 560 responses (28 participants × 10 sentences × two conditions) in Tasks 1 and 3, respectively, and 280 (28 participants × 10 sentences) responses in Task 2 were collected. We first compared the mean accuracy score of each task, and then used logistic mixed-effects model in R to analyze the differences in accuracy score in Tasks 1 and 3.

## Results

### Participants’ Overall Accuracy Rate in Each Task

**Figure 3:**
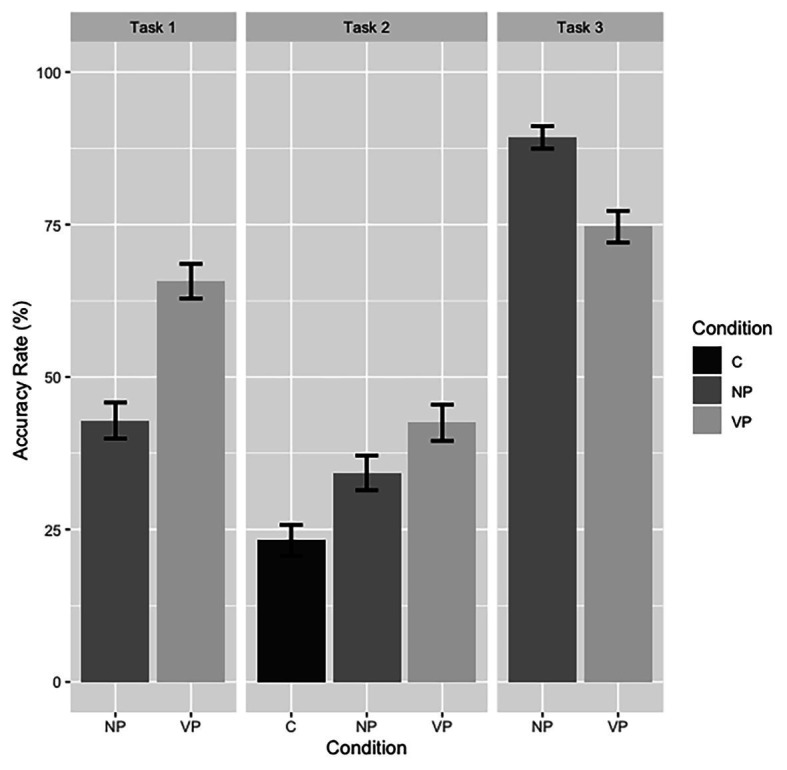
Accuracy rate for Tasks 1–3.

The accuracy rates for Tasks 1–3 are summarized in Figure 3. For Task 2, we used “Condition” instead of “Preference” in the X-coordinate, and “Accuracy Rate” rather than “Percent” in Y-coordinate so that Tasks 1–3 can be compared in the same figure. Therefore, in Tasks 1 and 3, “VP” and “NP” reflect subjects’ accurate understanding of the sentences when prosody supports VP-attachment and NP-attachment, respectively. Whereas, in Task 2, when prosodic cues were not provided, “VP” and “NP” represent participants’ preferred VP-attachment and NP-attachment of the ambiguous sentences, and “C” shows their analysis that the sentences were ambiguous. The error bars represent the stand error.

It can be observed that in Task 1 when participants heard the ambiguous sentences, their accuracy rates for the VP-attachment and NP-attachment are 65.7 and 42.9%, respectively, indicating their preference for the VP-attachment. When prosodic cues were not provided in Task 2, the percentage that the sentence is perceived to be ambiguous is only 23.2%. However, in Task 3, participants showed a bias toward the NP-attachment (82%) after they were informed of the ambiguity, and their overall accuracy rate improved a lot as well (74.6% for the VP-attachment). This finding suggests that participants could employ prosodic cues to identify the intended meaning of the ambiguous sentences after they were informed of the ambiguity. Results in Tasks 1 and 2 indicate that their failure to disambiguate the sentences may result from their unawareness of the ambiguity within this structure rather than their inability to detect prosodic cues.

To further examine the differences in the accuracy rates between Tasks 1 and 3, we analyzed the data with R (Version 1.1.463; Bates et al., 2014). Implemented with “glmer” function (binomial family: correct vs. incorrect) in the lme4 package in R, Generalized Linear Mixed model included participants’ response as dependent variable: correct (coded as 1) and incorrect (coded as 0), task (categorical predictor: Task 1 coded as 1 and Task 2 as −1), condition (categorical predictor: VP-attachment coded as 1 and NP-attachment as −1), and the interaction of task and condition as fixed effects. Participant and sound items were entered as random intercepts. The results are displayed in Table 1.

**Table 1:** Mixed-effects model for accuracy rate in Tasks 1 and 3.

Fixed effects	Estimate	*SE*	*z*-value	*p*
Intercept	0.927	0.119	7.799	^***^
Task	−0.736	0.075	−9.759	^***^
Condition	0.020	0.095	0.209	0.835
Task* condition	−0.512	0.075	−6.826	^***^

^***^ *p* < 0.001.

A main effect of task and the interaction of task and condition were revealed. The significant effect of task indicates a significantly higher accuracy rate for Task 3 than Task 1. The significant interaction between task and condition suggests a significantly lower accuracy rate in the NP-attachment in Task 1 and a significantly higher NP-attachment in Task 3. Given the significant interaction effects between task and condition, we conducted Tukey’s *post-hoc* comparisons with emmeans function in R (Lenth, 2018) to compare the accuracy rate in each condition within and across each task. The results, as shown in Table 2, were given on log odds ratio scale.

**Table 2:** *Post-hoc* pairwise comparisons for accuracy rate in Tasks 1 and 3.

Contrast	Estimate	*SE*	*z*-ratio	*p*
T1 NP–T1 VP	−0.985	0.214	−4.608	^***^
T3 NP–T3 VP	1.064	0.267	3.988	^**^
T1 NP–T3 NP	−2.496	0.233	−10.702	^***^
T1 VP–T3 VP	−0.448	0.190	−2.357	0.086

^**^ *p* < 0.05; ^***^ *p* < 0.001.

According to Table 2, L2 learners showed a significantly higher accuracy rate for the VP-attachment than NP-attachment in Task 1 and a significantly higher accuracy rate for the NP-attachment than VP-attachment in Task 3. Compared with Task 1, their accuracy rate for the NP-attachment was significantly improved in Task 3.

In Tasks 1 and 2 before participants received specific information about syntactic ambiguity, they did not realize that there might be two alternative interpretations for one structure, and showed a preference for the VP-attachment to NP-attachment. After they were informed of the ambiguity, however, they shifted their parsing preference and showed a bias toward the NP-attachment. Results of the overall accuracy rate averaged across participants revealed that L2 learners could employ prosodic information to resolve syntactic ambiguity, but they tended to pay more attention to the syntactic structure than prosodic information after detecting the ambiguity.

### Individual Participants’ Accuracy Rate in Each Task

The accuracy rate of individual participants in each task was further examined to see to what extent the learners differed in their ability to disambiguate sentence meanings. Table 3 summarizes the distribution of individual participants’ accuracy rate, and Table 4 presents each participant’s accuracy rate for each condition in each task. For Tasks 1 and 3, “VP” and “NP” represent participants’ accuracy rates when prosody supported VP-attachment and NP-attachment, respectively. For Task 2, “Ambiguous,” “VP,” and “NP” represent participants’ interpretation of the ambiguous sentence when prosodic cues were not provided. Table 3 reveals considerable variability that some participants were quite accurate and others were poor. In Task 1, the number of participants distributed within 76–100% range was the most in VP-attachment. But in NP-attachment, most of the participants distributed within 0–25% range. In Task 2, only five participants judged the sentences to be ambiguous, and the majority were distributed within 0–25% range. By comparison, their accuracy rate in Task 3 was most distributed within 76–100% for both the VP-attachment and NP-attachment, even though there were still some participants who could not effectively utilize prosodic information. It can be seen from Table 4 that in Task 1, some participants were quite accurate in VP-attachment, while others were quite accurate in NP-attachment. However, after they were informed of the ambiguity in Task 3, some participants shifted their preference, while others still had a bias or reversed their bias from one interpretation toward the other. Therefore, the analysis of individual participants’ accuracy rate revealed individual differences in parsing strategy, indicating an inconsistent parsing pattern among the learners.

**Table 3:** Frequency distribution of participants’ accuracy rate in each task.

Task							
Range of accuracy rate (%)	1		2			3	
	VP	NP	Ambiguous	VP	NP	VP	NP
0–25	3 (10.71%)	12 (42.86%)	20 (71.43%)	14 (50%)	14 (50%)	3 (10.71%)	1 (3.57%)
26–50	6 (21.43%)	6 (21.43%)	2 (7.14%)	1 (3.57%)	8 (28.57%)	2 (7.14%)	0 (0%)
51–75	6 (21.43%)	3 (10.71%)	1 (3.57%)	4 (14.29%)	1 (3.57%)	4 (14.29%)	2 (7.14%)
76–100	13 (46.43%)	7 (25%)	5 (17.86%)	9 (32.14%)	5 (17.86%)	19 (67.86%)	25 (89.29%)

**Table 4:** Accuracy rate of each participant in each task (%).

Task							
Participant	1		2			3	
	VP	NP	Ambiguous	VP	NP	VP	NP
1	100	0	50	0	50	100	100
2	40	100	0	30	70	30	90
3	40	40	10	60	30	100	10
4	10	90	0	0	100	70	60
5	70	10	0	90	10	90	80
6	90	10	0	0	100	80	100
7	80	50	80	0	20	100	80
8	80	0	100	0	0	80	80
9	40	70	0	100	0	90	90
10	90	10	0	50	50	100	90
11	70	50	100	0	0	70	100
12	70	40	0	60	40	100	80
13	60	40	0	20	80	100	100
14	80	100	90	0	10	90	100
15	100	0	0	90	10	60	70
16	90	10	0	80	20	0	100
17	80	20	60	0	40	100	100
18	20	90	50	20	30	0	100
19	60	80	0	80	20	40	90
20	60	80	0	10	90	60	100
21	100	0	0	70	30	90	100
22	20	80	0	0	100	100	100
23	30	90	90	0	10	80	100
24	50	50	0	100	0	80	100
25	80	10	10	90	0	90	80
26	30	70	10	60	30	90	100
27	100	0	0	80	20	80	100
28	100	10	0	100	0	20	100

## Discussion and Conclusion

This study employed both listening and reading tasks to probe into Chinese L2 English learners’ use of prosody to resolve syntactic ambiguity. Our primary purpose is to decide what results in L2 learners’ failure to utilize prosodic cues in L2 syntactic ambiguity resolution and in particular to examine the effect of ambiguity awareness. Although the three tasks in this study employed the same stimuli, which might exert some effects on the learners’ ambiguity awareness and their use of prosody, but this would not affect the conclusion. Firstly, although some of the participants have reported their ambiguity awareness before the brief interview, other participants were informed of the ambiguity in the brief interview. Therefore, all participants understood the ambiguity within the structure in Task 3. Secondly, we did not provide the participants with any feedback on how to disambiguate these sentences with prosody through the whole experiment. In addition, only Tasks 1 and 3 involved prosodic information, but Task 1 was completed in the 1st week, and Task 3 was conducted 1 week later so that the repetition effects were kept minimum. Moreover, with a focus on the effect of syntactic ambiguity, this study tested the same participants with the same stimuli in the whole experiment, which could help us better compare the results in different tasks.

The analysis of the overall accuracy rate reveals that before receiving specific instruction about syntactic ambiguity, when participants listened to the ambiguous sentences in Task 1 and read these sentences in Task 2, they preferred the VP-attachment. The VP-attachment of the PP-attachment ambiguity can be explained by the Minimal Attachment principle, which proposes that the parser builds the sentence structure in a simplest way (Frazier and Fodor, 1978). This finding provides a clear demonstration that prosodic cues with disambiguating information cannot effectively reduce the learners’ parsing bias. Our data in Task 2 show that most of the participants did not detect the syntactic ambiguity within the structure. In the brief interview with the participants, one of them reported his ambiguity awareness in Task 1, and four additional participants noticed the ambiguity in Task 2, but others reported that they had never come across such a structure before the experiment and that they did not detect the alternative interpretations for one sentence. It can be observed in Table 3 that the one (Participant 14) who reported his awareness of ambiguity in Task 1 performed much better than the others. It provides further evidence that participants’ failure to use prosodic cues in Task 1 may result from their lack of syntactic ambiguity awareness, instead of their inability to detect prosodic cues. To avoid a reanalysis of the sentence which requires more cognitive resource, they adopted a “good-enough” heuristic to interpret the sentence structure quickly and efficiently, regardless of the available disambiguating prosodic cues (Ferreira et al., 2002). Although participants’ preference for the VP-attachment before receiving specific information about ambiguity is the same as that of the native speakers in reading tasks (Britt, 1994; Spivey-Knowlton and Sedivy, 1995), it does not necessarily indicate a native-like parsing strategy by L2 learners, because the lack of ambiguity awareness constrained their conscious processing, and they tended to construct a single interpretation. Whereas, the native speakers are more likely to build both interpretations in parallel and weight the VP-attachment more than the NP-attachment, as the parallel parsing model predicts.

To further test this assumption, we explained to them about the syntactic ambiguity, but did not provide any prosodic strategy to resolve the ambiguity or any correct answer to the experimental items. After they understood syntactic ambiguity and had a rest, Task 3 was conducted. Compared with Task 1, the overall accuracy rate for both the VP-attachment and NP-attachment in Task 3 was significantly improved. This result further showed that participants’ failure to resolve ambiguity in Task 1 may be attributed to their lack of ambiguity awareness. However, in Task 3, the accuracy rate for the NP-attachment was significantly higher than that for the VP-attachment, indicating an over interpretation of the NP-attachment. It is assumed that after L2 learners had received specific information about syntactic ambiguity, they paid much more attention to the less frequent NP-attachment than the disambiguating prosodic cues to avoid incorrect interpretation. This result suggests that the learners’ analysis of the sentence structure is more syntactically than prosodically driven, contradicting to the SSH. However, this does not indicate their low sensitivity to prosodic cues, because previous studies have reported that both Chinese and English listeners weighted pause more heavily than final lengthening or pitch reset in speech perception (Zhang, 2012; Yang et al., 2014), and our speech production study has shown that all our stimuli have been correctly disambiguated with pause (Zhang et al., 2018). Furthermore, the participants’ accuracy rate was low for merely one interpretation rather than for both interpretations. If the low accuracy rate was attributed to the learners’ low sensitivity to prosodic cues, then they would have the same difficulty to determine the other interpretation, which contradicts with their performance. Therefore, we argue that the participants’ preference for the NP-attachment may come from the fact that they had difficulty in integrating prosodic information to syntactic structure properly, as has been suggested in the Interface Hypothesis (Ying, 1996; Sorace and Serratrice, 2009; Nakamura et al., 2016, 2020).

The analysis of the individual accuracy rate reveals considerable individual variability. Participants in Task 1 were quite accurate in one of the interpretations, but were poor in the other one, indicating that they could not utilize prosodic cues to resolve syntactic ambiguity efficiently. In Task 2, only five of them judged the sentences to be ambiguous, and the others preferred either the VP-attachment or NP-attachment. Furthermore, some of the participants reversed their parsing bias from one interpretation in Task 1 toward the other one in Task 2, indicating that they might be not quite sure about their response in Task 1 and guessed the meaning of the sentences. It is thus suggested that L2 learners’ lack of ambiguity awareness constrains their full analysis of the structure in Tasks 1 and 2. Participants’ preference for the VP-attachment in unconscious processing can be explained by the Minimal Attachment principle. But other participants’ preference for the NP-attachment suggests that Minimal Attachment principle cannot always predict L2 learners’ parsing performance, and their parsing strategy, which is related to their linguistic experience, may have to be learned anew (Cutler, 2012; Ip and Cutler, 2018). That most of the participants’ bias was reduced after they were informed of the ambiguity in Task 3 suggests that the learners could use prosodic cues to resolve ambiguity. However, there were still some participants who could not shift their original parsing bias or reversed their bias from one interpretation toward the other one. We have argued that this may come from their difficulty in integrating prosodic information to syntactic structure (Roberts et al., 2008; Sorace and Serratrice, 2009; Nakamura et al., 2016, 2020).

Given the above findings, the fact that most of the L2 learners were able to utilize prosodic cues after they were informed of the ambiguity suggests that their failure to resolve ambiguity in the previous tasks can be attributed to their unawareness of syntactic ambiguity that constrains their full analysis of the ambiguous structure. However, the finding that some participants reversed their parsing bias from one interpretation toward the other one cannot be simply generalized to a lack of ambiguity awareness. The analysis of the individual accuracy rate indicates the individual variability in L2 learners’ parsing pattern. For instance, the learners’ lack of ambiguity awareness may constrain a full analysis of the complex structure, regardless of the parsing strategy. In addition, they may experience difficulty in integrating prosodic information to syntactic structure properly, because prosodic and syntactic structures are not always isomorphic (see Shattuck-Hufnagel and Turk, 1996; Cutler et al., 1997, for a review). Moreover, the Interface Hypothesis proposes that when sentence processing involves an integration of multiple information sources, and L2 learners experience more difficulties, which increases the computational burden within the limits of cognitive resources (Fultz, 2008; Sorace and Serratrice, 2009). Spoken language comprehension is further burdened by the transient nature of the speech signal, and they cannot refer back to the prosodic information after listening. Therefore, it is suggested that the learners’ failure to use prosodic information can be attributed to their lack of ambiguity awareness and their difficulty in information integration.

The experiment reported here also provides theoretical implications for L2 research. It has been previously argued in SSH that L2 learners rely more on lexical, semantic, and pragmatic rather than syntactic information (Papadopoulou and Clahsen, 2003; Papadopoulou, 2005; Clahsen and Felser, 2006a,b). The participants’ consistent preference for the VP-attachment before being informed of the ambiguity in Tasks 1 and 2 does not necessarily indicate that they can acquire the native-like parsing strategy, because their unconscious processing constrains a full analysis of the structure by building only one interpretation. Therefore, their preference for the VP-attachment does not result from weighting VP-attachment more than NP-attachment as the native speakers do. Whereas, the participants’ preference for the NP-attachment after being informed of the ambiguity in Task 3 suggests that they pay more attention to the syntactic structure than to the prosodic information, indicating a more structure-driven parsing strategy. This contradicts to the SSH and suggests that L2 learners’ parsing strategy is not “shallower” than that of the native speakers, although their difficulty in information integration leads to a preference for the NP-attachment to the VP-attachment interpretation. The individual parsing performance shows that the learners’ parsing strategy is related to their linguistic experience, and the Minimal Attachment principle cannot always predict the learners’ parsing preference. That some participants failed to resolve syntactic ambiguity with prosodic cues may suggest that their inability to integrate prosodic information to syntactic structure efficiently, as has been proposed in the Interface Hypothesis (Sorace and Serratrice, 2009).

Recent decades have witnessed an increasing number of studies investigating how L2 learners process ambiguous sentences in spoken language comprehension. Some of the earlier studies have reported that L2 learners have difficulty in ambiguity resolution in spoken language comprehension, which may be attributed to their low sensitivity to prosodic cues, low L2 proficiency, difficulty in information integration, L1 background, limited learning experience, and limited cognitive resources, or that the L2 parsing strategy has to be learned anew (Harley et al., 1995; Ying, 1996; Dekydtspotter et al., 2008; Nakamura et al., 2016; Ip and Cutler, 2018; Nickels and Steinhauer, 2018). Our present study provides additional evidence that L2 learners’ difficulty in prosodic disambiguation may come from their lack of ambiguity awareness that constrains them to detect the ambiguity within the structure. In this situation, the learners interpret the ambiguous sentences according to their learning experience in a “good-enough” processing heuristic to avoid more computational burden (Ferreira et al., 2001, 2002; Christianson et al., 2006; Ferreira and Patson, 2007; Slattery et al., 2013; von der Malsburg and Vasishth, 2013; Christianson, 2016). Their failure to resolve syntactic ambiguity with disambiguating prosodic information efficiently after being informed of the ambiguity indicates the individual variations in information integration rather than their low sensitivity to prosodic cues. By employing the spontaneously produced stimuli by different speakers, we find that L2 learners have difficulty in using prosody to resolve syntactic ambiguity. This difficulty is likely coming from their lack of ambiguity awareness and their difficulty in information integration, instead of a low sensitivity to prosodic cues or inability to detect prosodic cues.

## Data Availability Statement

The raw data supporting the conclusions of this article will be made available by the authors, without undue reservation.

## Ethics Statement

The studies involving human participants were reviewed and approved by Ethic Committee of School of Foreign Languages, Shanghai Jiao Tong University. The patients/participants provided their written informed consent to participate in this study.

## Author Contributions

YZ and HD conceived and designed the experiment. YZ created the stimuli, programed the experiment, recruited and tested participants, collected the data, conducted data analyses, and wrote the manuscript. HD provided critical feedback during the whole process, and revised the manuscript. All authors contributed to the article and approved the submitted version.

### Conflict of Interest

The authors declare that the research was conducted in the absence of any commercial or financial relationships that could be construed as a potential conflict of interest.
